# Development of an *in-vitro* model for extracorporeal blood pumps to study the effects of artificial pulsatility on human blood

**DOI:** 10.3389/fmed.2023.1237002

**Published:** 2023-08-28

**Authors:** Barbara Zieger, Denise Schneider, Sam Joé Brixius, Christian Scherer, Armin Buchwald, Georg Trummer, Martin Czerny, Friedhelm Beyersdorf, Hans-Jörg Busch, Christoph Benk, Jan-Steffen Pooth

**Affiliations:** ^1^Department of Pediatrics and Adolescent Medicine, University Medical Centre Freiburg, Faculty of Medicine, University of Freiburg, Freiburg, Germany; ^2^Department of Cardiovascular Surgery, University Medical Centre Freiburg, Faculty of Medicine, University of Freiburg, Freiburg, Germany; ^3^Institute for Clinical Chemistry and Laboratory Medicine, University Medical Centre Freiburg, Faculty of Medicine, University of Freiburg, Freiburg, Germany; ^4^Department of Emergency Medicine, University Medical Centre Freiburg, Faculty of Medicine, University of Freiburg, Freiburg, Germany

**Keywords:** extracorporeal circulation, pulsatility, diagonal pumps, *in-vitro*, *ex-vivo*, acquired von Willebrand syndrome, hemolysis, platelet activation

## Abstract

**Introduction:**

The application of extracorporeal circulation (ECC) systems is known to be associated with several implications regarding hemolysis, inflammation, and coagulation. In the last years, systems with pulsatile blood flow are increasingly used with the intention to improve hemodynamics in reperfusion. However, their implications on the aforementioned aspects remain largely unknown. To investigate the effects of pulsatility, this *ex-vivo* study was initiated.

**Methods:**

Test circuits (primed with human whole blood) were set up in accordance with the recommendations of international standards for *in-vitro* evaluation of new components and systems of ECC. Diagonal pumps were either set up with non-pulsatile (n = 5, NPG) or pulsatile (n = 5, PG) pump settings and evaluated for 6 h. All analyses were conducted with human whole blood. Blood samples were repeatedly drawn from the test circuits and analyzed regarding free hemoglobin, interleukin 8 (IL-8), platelet aggregation and acquired von Willebrand syndrome (AVWS).

**Results:**

After 1 h of circulation, a significant coagulation impairment (impaired platelet function and AVWS) was observed in both groups. After 6 h of circulation, increased IL-8 concentrations were measured in both groups (NPG: 0.05 ± 0.03 pg./mL, PG: 0.03 ± 0.01 pg./mL, *p* = 0.48). Pulsatile pump flow resulted in significantly increased hemolysis after 6 h of circulation (NPG: 37.3 ± 12.4 mg/100 L; PG: 59.6 ± 14.5 mg/100 L; *p* < 0.05).

**Conclusion:**

Our results indicate that the coagulative impairment takes place in the early phase of ECC. Pulsatility did not affect the occurrence of AVWS *ex-vivo*. Prolonged durations of pulsatile pump flow led to increased hemolysis and therefore, its prolonged use should be employed cautiously in clinical practice with appropriate monitoring.

## Introduction

In recent years and especially during the COVID-19 pandemic, there has been an increase in the use of extracorporeal membrane oxygenation (ECMO) systems and also an increase in the number of centers that can offer ECMO support (1, 2). However, despite their increasing application the use of extracorporeal circulation (ECC) is still associated with several implications regarding hemolysis, inflammation, and impaired coagulation (3–5). In the continuous development of system components of ECC, the optimization of these implications has been constantly pursued. The underlying pathomechanisms are multifactorial (6). On one hand, they include device-associated and thus hardly alterable factors such as the contact of blood with foreign surfaces and blood exposure to increased shear stress in the area of the pump head (7). On the other hand, there are also factors such as non-physiological perfusion caused by a non-pulsatile flow and in case of venoarterial cannulation retrograde perfusion of organs.

Modern systems for extracorporeal membrane oxygenation now offer the possibility to generate pulsatile flow and thus a pulsatile and potentially more physiological tissue perfusion thanks to diagonal pump technology. These systems include, for example, the i-cor^®^ system (Xenios AG, Heilbronn, Germany) and the CARL^®^ controller (Resuscitec GmbH, Freiburg, Germany). By generating pulsatile flow, physiological hemodynamic energy levels can be achieved to improve microcirculation (8). Various animal studies have already been able to demonstrate that the application of a pulsatile extracorporeal flow achieves higher energy equivalent pressures and hemodynamic energies which ultimately result in an improvement of microcirculation (9).

This seems to be desirable especially in the context of a resuscitation situation after prolonged cardiac arrest. Our group has already been able to show superior survival with the use of controlled pulsatile perfusion in an animal model (10). Furthermore, first reports on the clinical application of controlled, pulsatile reperfusion also present promising results (11).

However, it is unclear to what extent a pulsatile and therefore physiological flow also affects and possibly positively influences device-associated factors of ECC. Therefore, to investigate the effects of pulsatility on hemolysis, inflammation, and coagulation, this *ex-vivo* study was initiated.

## Methods

This study was approved by the Ethics Committee of the Albert-Ludwigs-University Freiburg (Ethics Committee Number 497/19) and registered with the German Clinical Trials Register (www.drks.de/DRKS00017402). Written consent was obtained from all participants in accordance with the Declaration of Helsinki.

**Figure 1:**
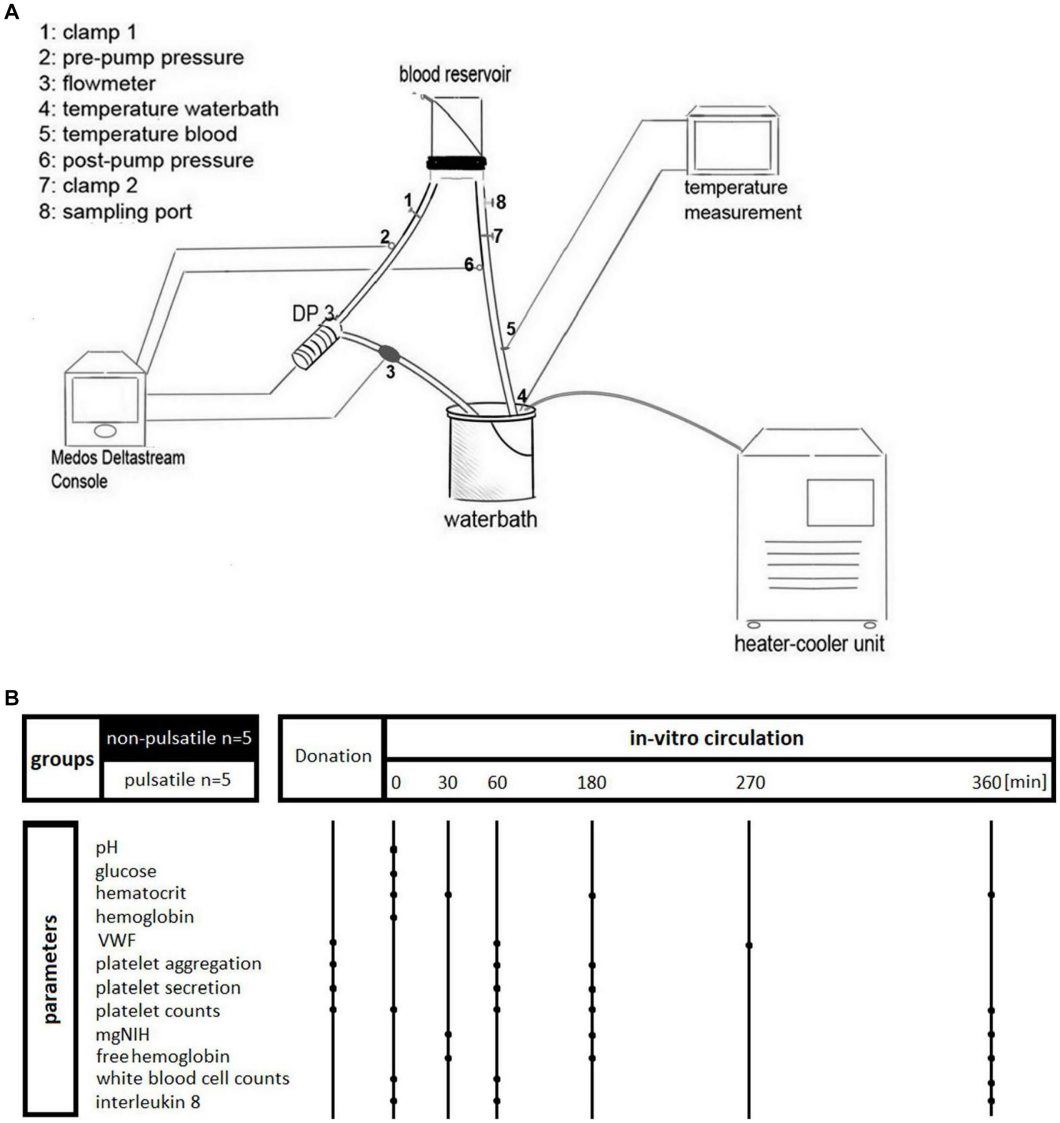
**(A)** Schematical depiction of the experimental set-up. **(B)** Overview of the experimental groups and study parameters.

The experimental set-up is depicted in Figure 1. Test circuits were set up in accordance with the recommendations of international standards for *in-vitro* evaluation of new components and systems of ECC (12–14). Each circuit contained a diagonal pump (Pump Head Medos Deltastream^®^ DP 3, Xenios AG, Heilbronn, Germany), connecting tubing (Polyvinylchlorid tube 3/8 × 3/32, 2 m, Medos, Stolberg, Germany) and a custom-made, small size blood reservoir, adapted from the work by Olia et al. (15). For pressure monitoring one manometer was placed at the inlet of the pump and another at the outlet of the pump. Clamps were positioned to enable pressure regulation in the system. An ultrasonic flow meter was positioned at the pump outlet tubing. A water bath was used to regulate the temperature in the circuit. Both in the water bath and in the system, the temperature was monitored during the entire study period.

### Experimental procedure

First the test circuit was filled with 500 mL 0.9% saline to wet all surfaces. Blood was collected by venipuncture and drawn into a bag containing the stabilizing anticoagulant CPD-1 (CompoFlex^®^ Single System, Fresenius Kabi Austria GmbH, Graz, Austria). Within 30 min of blood collection, the test circuit was drained completely and then refilled with 300 mL human whole blood and 60 mL of 0.9% saline. In one group (*N* = 5), the pump was set to a non-pulsatile (NP) flow, while in the other group (*N* = 5) pulsatile (P) pump settings were used. Each pump ran for 6 h at 37°C with an inlet pressure of −30 mmHg, an outlet pressure of 300 mmHg and a total blood flow of 6.5 L min^−1^. The flow rate was monitored continuously. In the NP group, the pumps were run with a fixed setting of 8,000 rotations per minute (rpm). For the P group, pump settings were chosen to achieve a pulse frequency of 50 min^−1^, a pump speed difference of 2,500 rpm and a pressure amplitude of 50 mmHg following the clinical practice of extracorporeal cardiopulmonary resuscitation.

### Blood sampling

All measured parameters and their time of measurement are shown in Figure 1B. At the time of venipuncture (baseline) additional blood was drawn for selected parameters (VWF analysis, platelet aggregometry analyses, CD 62, CD 63). Further blood samples were repeatedly drawn from the circuit at the start of circulation, after 30 min, 1 h, 3 h, 4.5 h and 6 h of circulation, respectively. The blood samples were stored at room temperature and processed within the first hour of collection.

### Hemostaseological parameters

Von Willebrand factor (VWF) antigen (VWF:Ag), VWF:collagen binding activity (VWF:CB) and VWF multimers were determined as previously described (16–18). The ratios of VWF:CB/VWF:Ag (normal ≥0.7) were calculated in order to reflect the biological capacity of the available VWF to bind to collagen. Acquired Von Willebrand syndrome (AVWS) were diagnosed if the ratio VWF:CB/VWF:Ag was reduced (<0.7) and if the high molecular weight multimers (HMW) were missing (16–18). Platelet aggregregometry was performed as described previously by our group using the aggregometer APACT 4.0 (BioMedical Technologies, Ahrensburg, Germany) and the agonists ADP (4 μmol/L), collagen (2 μg/mL), epinephrin (8 μmoL/L), or ristocetin (1.2 mg/mL) (17, 19).

The analysis of platelet granule secretion was performed using flow cytometry. Platelets were stimulated with increasing concentrations of thrombin and incubated with fluorescently labeled CD62 (for α-granules) and CD63 (for δ-granules) antibodies using an established protocol (7, 20).

### Measurement of hemolysis

Free hemoglobin was measured by photometry. Hematocrit, hemoglobin, pH and glucose were analyzed using a mobile blood gas analysis system (i-STAT 1, Abbott GmbH, Wiesbaden, Germany). For a standardized comparison of hemolysis the normalized milligram index of hemolysis (mgNIH) was calculated (14). The index is defined as free hemoglobin in mg released from 100 L of circulating blood (14).

### Measurement of interleukin 6 and 8

To analyze the inflammatory response the interleukins 6 (IL-6) and 8 (IL-8) were measured by a multiplex bead assay (Bio-Plex Pro Cytokine, Chemokine, and Growth Factor Assays, Bio Rad Laboratories GmbH, Feldkirchen, Germany). The measurements were performed according to the manufacturer’s instructions (21).

### Statistical analysis

All statistical analyses and visualizations were performed using RStudio (version 4.0.3, Boston, Massachusetts, United States) (22). Students t-tests were used in case of normal distribution and variance homogeneity, otherwise Mann–Whitney-U test were applied for multiple comparisons to assess the changes in absolute cell numbers, hemostaseological parameters and IL-8 concentration over time between pulsatile and non-pulsatile pump flow. Differences regarding platelet granule secretion were calculated using a two-way analysis of variance (ANOVA) test with repeated measures or, in order to examine the influence of group allocation, a three-factor ANOVA test followed by a paired t-test with Bonferroni correction as a *post hoc* test. In case of unequal group size, an unpaired t-test was used.

## Results

Table 1 shows the characteristics of blood donors and baseline measurement values. At the time of donation, no significant differences between the two groups were observed.

**Table 1:** Characteristics of blood donors.

	NP (*n* = 5)	P (*n* = 5)	*p*-value
Gender [Male/Female]	5/0	5/0	–
Age [years]	31 ± 13	33 ± 12	0.45
Hematocrit [%]	43.9 ± 4.2	44.7 ± 1.5	0.87
pH	7.37 ± 0.03	7.38 ± 0.03	0.29
Glucose [mg dl^−1^]	92 ± 10	93 ± 12	0.84
Hemoglobin [g dl^−1^]	14.8 ± 1.3	15.1 ± 0.4	0.89

NP, non-pulsatile; P, pulsatile.

### AVWS in all test circuits

**Figure 2:**
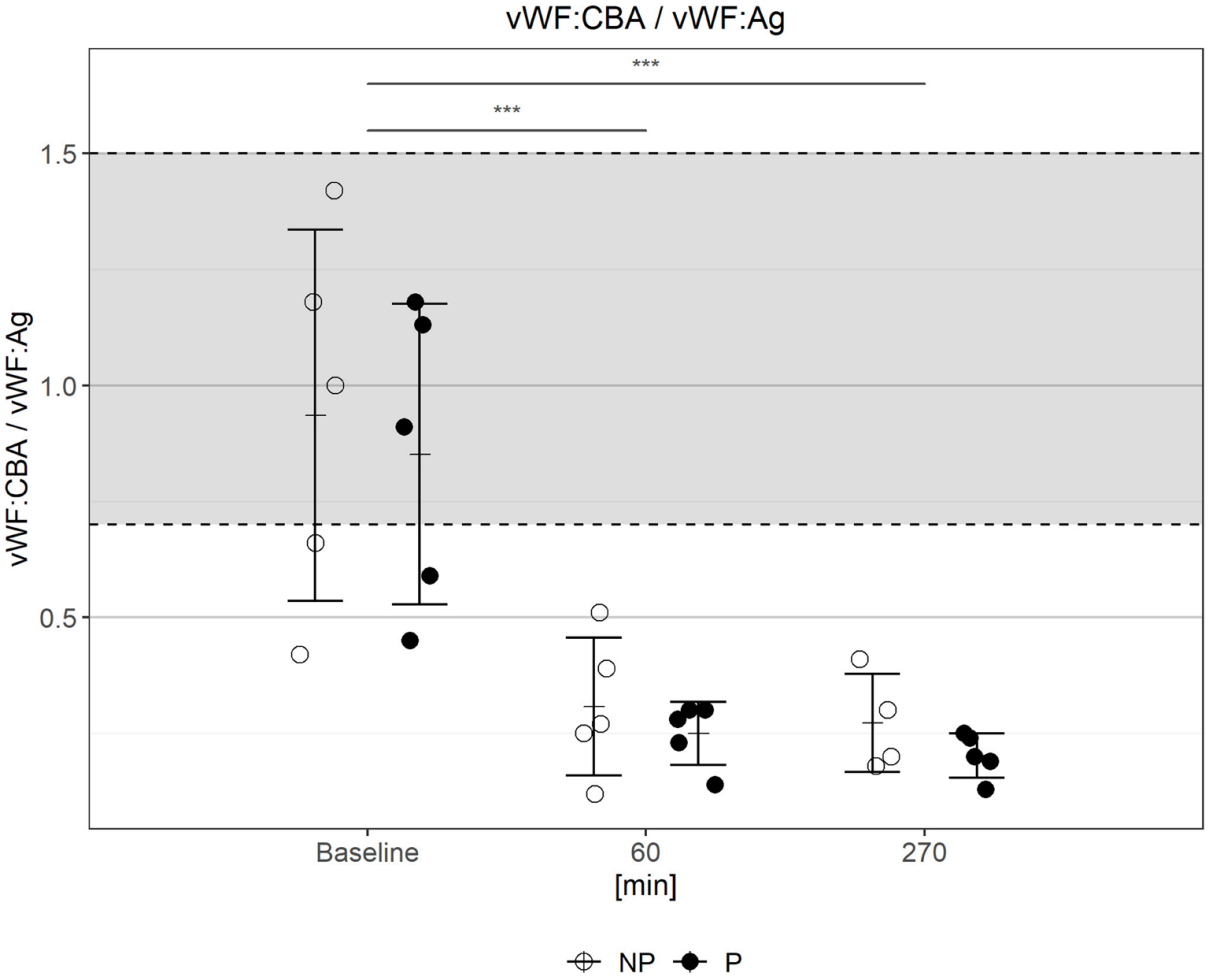
Ratio of VWF:CB to VWF:Ag during circulation. Gray area marks normal range. Bars depict mean ± standard deviation (sd). NP, non-pulsatile; P, pulsatile; ***p < 0.001.

The VWF:CB/VWF:Ag ratio decreased significantly over time in all experiments (Figure 2). After 60 min of *in-vitro* circulation every test circuit showed a pathological VWF:CB/VWF:Ag ratio (NP: 0.31 ± 0.15 vs. P: 0.25 ± 0.07, *p* = 0.45, Figure 2). Due to technical problems, no measurement could be obtained from one sample at 270 min in the non-pulsatile group. In all experiments a severe loss of VWF HMW-multimers was detected after 1 h of circulation (see Supplementary Figure 1). Therefore, all measurements during the *in-vitro* circulation phase met the criteria of an AVWS with no significant differences between the two groups at any time.

### Impaired platelet function

**Figure 3:**
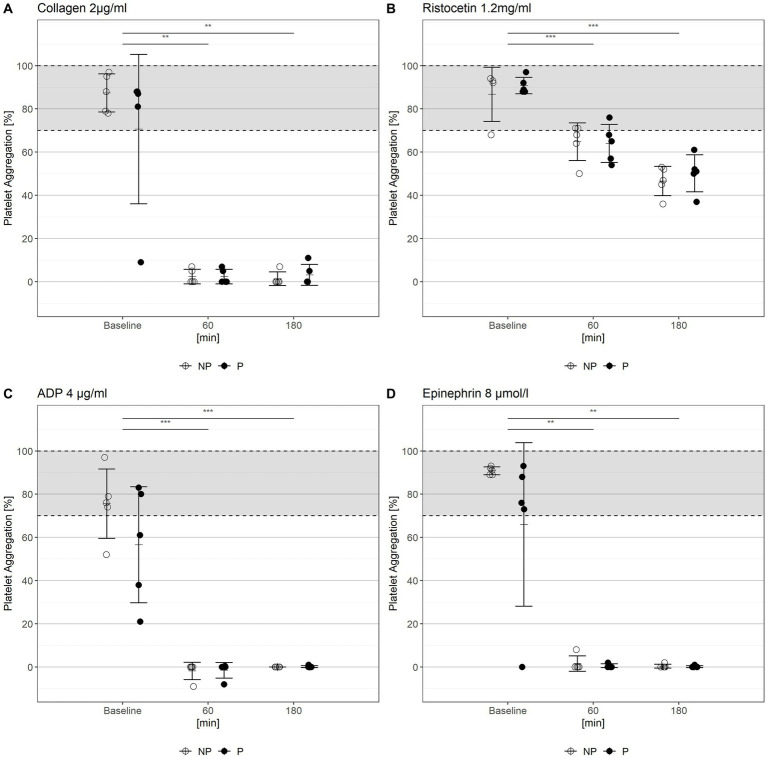
Platelet aggregation tests with **(A)** collagen, **(B)** ristocetin, **(C)** ADP, and **(D)** epinephrine as stimulating agents. Gray areas mark normal range. NP, non-pulsatile; P, pulsatile, ***p < 0.001, **p < 0.01.

The platelet aggregometry demonstrated a significant hypoaggregability in all experiments during the *in-vitro* circulation (Figure 3). Stimulation with collagen (Figure 3A), ADP (Figure 3C) and epinephrine (Figure 3D) resulted in an impaired platelet aggregation (maximum aggregation less than 10%) after 60 min of circulation (with no significant further decrease after 3 h). There were no significant differences between the two groups at any time.

**Figure 4:**
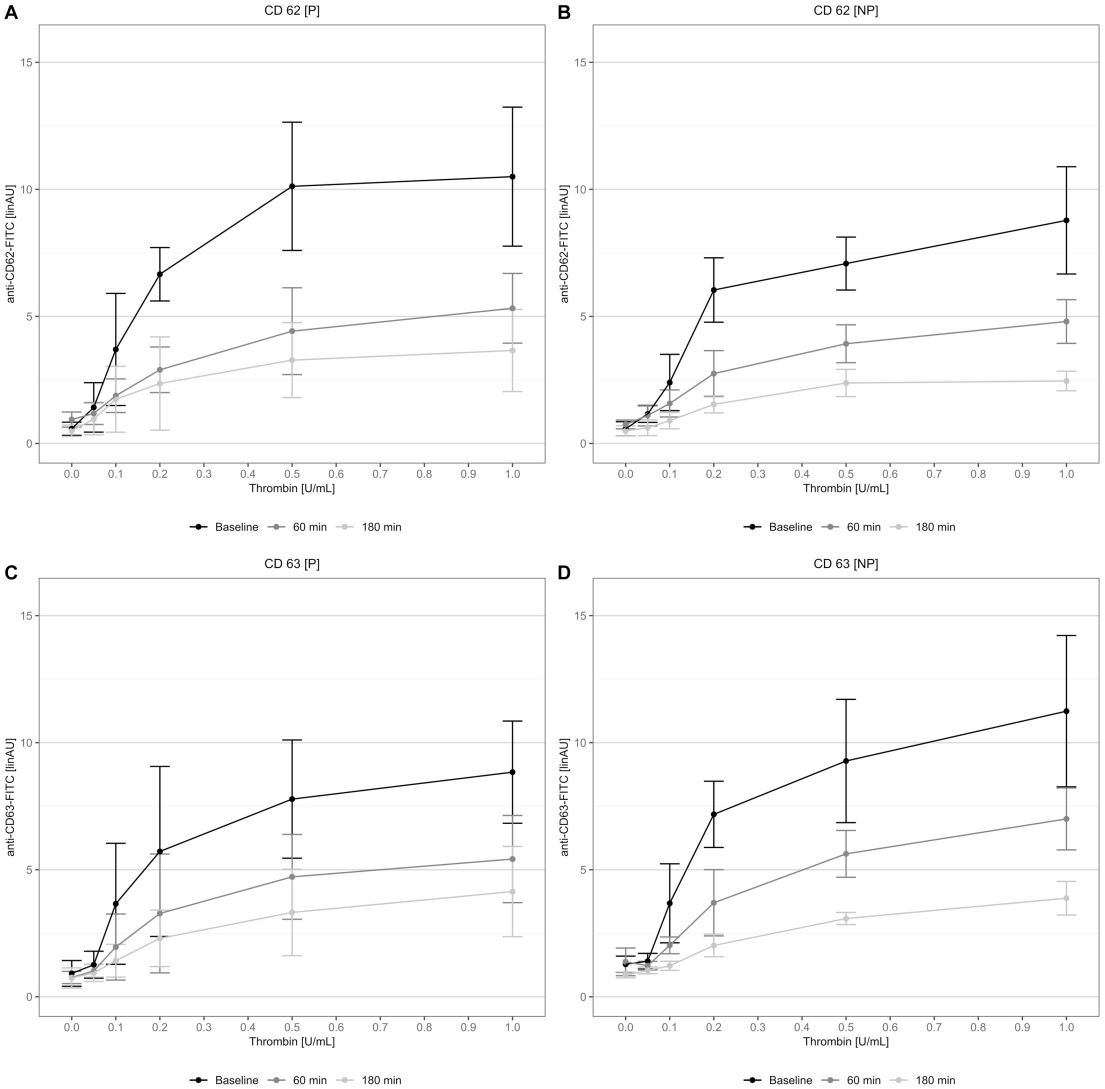
Platelet α-**(A,B)** and δ-granule **(C,D)** secretion stimulated with thrombin (0, 0.05, 0.1, 0.2, 0.5, and 1.0 U/mL) at the time of blood donation, after 60 and 180 min circulation. NP, non-pulsatile; P, pulsatile.

Likewise, the of platelet flow-cytometry analyses-revealed a significant reduced secretion of platelet α- and δ-granule after stimulation with thrombin in all measurements during the *in-vitro* circulation period (Figure 4). One measurement at 1 h of circulation from the non-pulsatile group could not be analyzed. There was a clear loss of granule secretion capacity when exposed to the extracorporeal circulation circuit, which aggravated over time. No significant differences could be shown between the two groups (compare Figures 4A vs. B and C vs. D).

### Reduced hemolysis under non-pulsatile conditions

**Figure 5:**
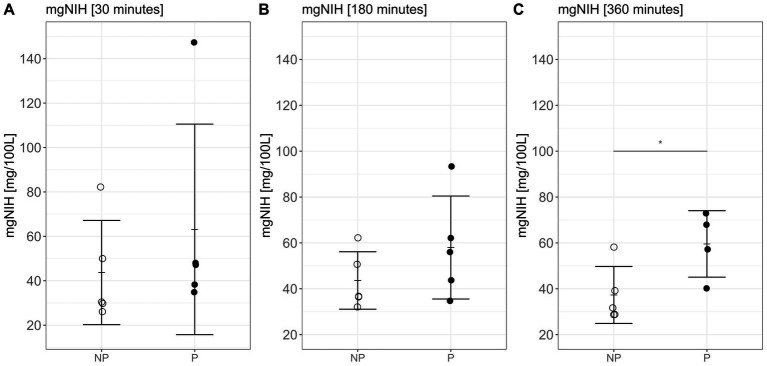
Normalized Milligram Index of Hemolysis (mgNIH) after **(A)** 30 min, **(B)** 180 min, and **(C)** 360 min of *in-vitro* circulation. Bars depict mean ± sd. NP, non-pulsatile; P, pulsatile, *p < 0.05.

Increased levels of free hemoglobin were detected in all experiments (Figure 5). Free hemoglobin could not be measured in one experiment at 360 min in the pulsatile group. A tendency toward a higher mgNIH in the pulsatile group could be detected at all time points. But this difference only proved to be significant after 360 min (after 360 min NP: 37.3 ± 12.4 mg/100 L; PG: 59.6 ± 14.5 mg/100 L *p* = 0.04, Figure 5C).

### No significant effect of pulsatility on inflammatory response

**Figure 6:**
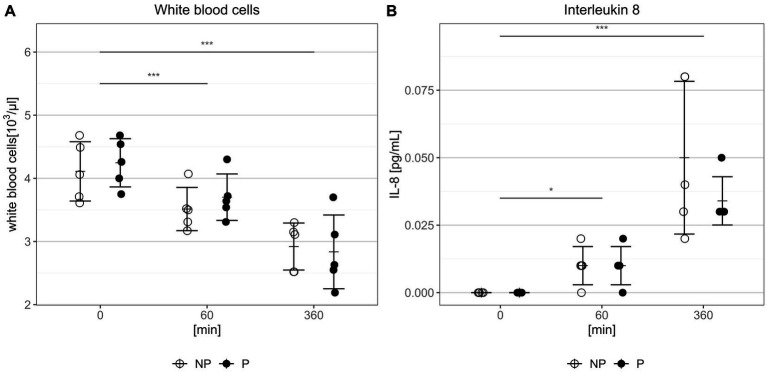
**(A)** Concentration of white blood cells at the beginning, after 60 min and after 360 min of circulation. **(B)** Concentration of interleukin 8 (IL-8) at the beginning, after 60 min and after 360 min of circulation. Bars depict mean ± sd. NP, non-pulsatile; P, pulsatile, ***p < 0.001, *p < 0.05.

The number of white blood cells decreased significantly over time in all experiments (after 360 min NP: 2.95 ± 0.34*103/μL vs. P: 2.84 ± 0.58*103/μL, *p* = 0.79, Figure 6A). No significant difference could be shown between the two groups. Levels of interleukin 6 fell continuously below the quantification limit of the chosen assay (<0,001 pg./mL) and were therefore excluded from the analysis. Meanwhile, interleukin 8 concentration increased significantly over the *in-vitro* circulation period (after 360 min NP: 0.05 ± 0.03 pg./mL vs. P: 0.03 ± 0.01 pg./mL, *p* = 0.48, Figure 6B). However, no significant differences between the two groups regarding interleukin 8 concentration were detected at any time.

## Discussion

The aim of this study was to investigate the isolated effect of pulsatility on hemolysis, inflammation, and coagulation on human whole blood *in-vitro*. Clinical studies proved a positive effect of pulsatility on microcirculatory blood flow and tissue oxygen saturation (23, 24). Pulsatile flow induces shear stress, which is responsible for NO release from the endothelium (25–27). NO exhibits various physiological functions, including inhibition of platelet aggregation, leukocyte adhesion, and vascular inflammation and it also exerts vasodilatory effects, thereby improving peripheral circulation (28). Moreover, the maintenance of pulsatile blood flow has shown a protective effect on the endothelial glycocalyx (29). However, it has also been shown that increased shear stress can have detrimental effects on red blood cells, ultimately leading to hemolysis (29). Our findings confirm increased hemolysis in prolonged durations of pulsatility. Throughout the period of *in-vitro* circulation, there was a notable trend toward heightened hemolysis under pulsatile conditions. After 6 h, this trend reached statistical significance, indicating a substantial difference in hemolysis between the pulsatile and non-pulsatile group (Figure 5). Our results suggest that hemolysis in artificial pulsatility may become more pronounced over time, highlighting the need for caution when utilizing pulsatile flow in extracorporeal circulation for extended periods. However, we did not investigate different parameters of pulsatility (e.g., frequency and amplitude) in relation to the resulting hemolysis. Furthermore, it is essential to acknowledge that the compensatory mechanisms in the human organism, particularly hepatic compensation for hemolysis, cannot be replicated in the selected *in-vitro* environment.

It is known that ECC is also associated with an impaired primary hemostasis (impaired platelet function and AVWS). Geisen et al., 2008 were the first to identify and describe that the exposure to increased shear stress in combination with contact to artificial surfaces leads to AVWS (16–18, 30). Kalbhenn et al. described for the first time AVWS in a large cohort of patients with ECMO support. In our study a loss of HMW (Supplementary Figure 1) combined with reduced VWF:CBA/VWF:Ag-ratio (Figure 2) was also observed in our model after 60 min of *in-vitro* circulation. Furthermore, an impaired platelet function (impaired α- and δ-granule-secretion, impaired aggregometry) was observed (Figures 3, 4). These observations have also been reported by our group in previous investigations in patients with ECC (17). AVWS and impaired platelet function increase the risk of bleeding, which is one of the most common complications associated with ECC (1). Mechanically-induced blood damage, contact with artificial surfaces, and the pump itself have all been identified as underlying mechanisms for this increased bleeding risk (31). Early detection and targeted therapy is critical in mitigating the risk of bleeding and improving outcomes for patients undergoing ECC (17). It is essential to note that our model only represented the isolated effects on the cellular and non-cellular components of the blood. The effects of pulsatility on the organism and the endothelium could not be taken into account. Vincent et al. demonstrated that pulsatility is a trigger for the endothelial release of VWF and that VWF degradation is modulated by pulsatility (32). These effects are impossible to be captured in an *ex-vivo* model.

When comparing two different methods of measuring the platelet count (impedance and fluorescence), a difference was identified between the two techniques. The scatterplots showed an increase in fragmentocytes, while the large-volume, immature platelets decreased (Supplementary Figure 2). This can be explained by the mechanical trauma. The shift of the maximum volumes to the left in the volume distribution curve also suggests a destruction of the larger cells, i.e., immature platelets. Using fluorescence measurements, the erythrocyte fragments are not erroneously measured along with the platelet fracture but are recorded as fragmentocytes. Accordingly, there were no false-high values for the platelets using fluorescence measurements. Supplementary Figure 3 demonstrates the differences between the two measurement methods and confirms the error-proneness of the impedance method. In the context of ECC and the associated high shear stress and cell destruction, careful consideration should be given regarding the choice of measurement method.

The use of ECC therapy can trigger an complex inflammatory response resulting in the activation of leukocytes (5). In their review article, Ki et al. reported on leukocytopenia in patients undergoing ECC and attributed it to adhesion of leukocytes to the artificial surface due to leukocyte activation (33). The reduction of white blood cells was also seen in our *in-vitro* study (Figure 6A). In addition to the observed leukocytopenia, our measurements showed an increase in interleukin-8 levels, consistent with previous reports in the literature (5). Nevertheless, the interleukin levels in our *ex-vivo* model were lower than in other studies (34). Differences in experimental design may contribute to this discrepancy, particularly the absence of an oxygenator in our setup. Previous studies have suggested that the oxygenator is a primary contributor to the inflammatory response associated with ECC therapy (35). This could be mainly due to the large surface area of the oxygenator. Furthermore, other studies have reported an elevation in cytokine levels, particularly after 6 h of ECC therapy. Since our study investigated a duration of only 6 h, the full impact of ECC on cytokine release may not have been fully captured (36).

In terms of pulsatility, no differences were observed in regard to leukocyte counts and interleukin 8 levels (Figure 6). However, it is known that pulsatility has a protective effect on the endothelial glycocalyx (29) and inhibits vascular inflammation through NO release (25, 26, 28). Therefore, it should be noted that the overall effects of pulsatility on the human inflammatory response are impossible to be captured in an *ex-vivo* model.

### Limitations of our study

While this *in-vitro* study provides further insights into the isolated effects of pulsatile and non-pulsatile flow on hemolysis, inflammation, and coagulation, there are some limitations to consider. First, the investigated duration of 6 h can be regarded as a limitation in view of considerably longer clinical applications of ECC. However, the chosen model settings were based on extreme settings according to international recommendations for *in-vitro* tests. This limits the direct translatability of the results into the clinical setting but should ensure comparability of our observations with other *in-vitro* tests. Secondly, as described in the methods section the investigated blood needed to be anticoagulated after venipuncture to avoid clotting during the set-up of the presented model. The anticoagulation was therefore regarded as unavoidable, and the authors are unaware of any implications regarding the investigated parameters.

## Conclusion

The presented model is suitable to mimic the effects of ECC on human whole blood *in-vitro*. The platelet function impairment and an AVWS seem to occur already in the very early phase of ECC even in the presence of pulsatility. Pulsatile flows did not lead to an attenuation of blood interleukin production *ex-vivo*. However, prolonged durations of pulsatility led to increased levels of hemolysis. Prolonged applications of pulsatility should therefore be used cautiously in clinical practice and be accompanied by appropriate monitoring.

## Data availability statement

The raw data supporting the conclusions of this article will be made available by the authors, without undue reservation.

## Ethics statement

The studies involving human participants were reviewed and approved by the Ethics Committee of the Albert-Ludwigs-University Freiburg. The patients/participants provided their written informed consent to participate in this study.

## Author contributions

BZ, DS, and J-SP planned and designed the study, and analyzed the data. DS, J-SP, SB, and CS conducted the *in-vitro* experiments. DS visualized the data and performed all statistical analyses. DS and J-SP drafted the original manuscript. AB, GT, H-JB, MC, CB, and BZ were involved in the interpretation of data and were major contributors in the revision of the manuscript. GT, CB, H-JB, FB, MC, and BZ provided clinical resources and funding. All authors read and approved the final manuscript.

## Funding

BZ and J-SP acknowledge funding by the Deutsche Forschungsgemeinschaft (DFG). No funding regarding this study was received. The authors acknowledge support by the Open Access Publication Fund of the University of Freiburg.

## Conflict of interest

FB is founder and shareholder and CB, and GT are shareholders of Resuscitec GmbH, Freiburg, Germany, which is a start-up company from the University Medical Centre Freiburg. CB, J-SP, SB and CS are part-time employees of Resuscitec GmbH, Freiburg, Germany.

The remaining author declares that the research was conducted in the absence of any commercial or financial relationships that could be construed as a potential conflict of interest.

## Publisher’s note

All claims expressed in this article are solely those of the authors and do not necessarily represent those of their affiliated organizations, or those of the publisher, the editors and the reviewers. Any product that may be evaluated in this article, or claim that may be made by its manufacturer, is not guaranteed or endorsed by the publisher.

## Abbreviations

AVWS: acquired von Willebrand syndrome
ECC: extracorporeal circulation
ECMO: extracorporeal membrane oxygenation
mgNIH: normalized milligram index of hemolysis
rpm: rotations per minute
VWF: Von Willebrand factor
