# Supplementary material for: Development of an in-vitro model for extracorporeal blood pumps to study the effects of artificial pulsatility on human blood
Source: Front Med (Lausanne). 2023 Aug 28;10:1237002. doi: 10.3389/fmed.2023.1237002 (PMC10497958; doi:10.3389/fmed.2023.1237002)
Supplement: Supplementary file 1 [file Data_Sheet_1.DOCX]

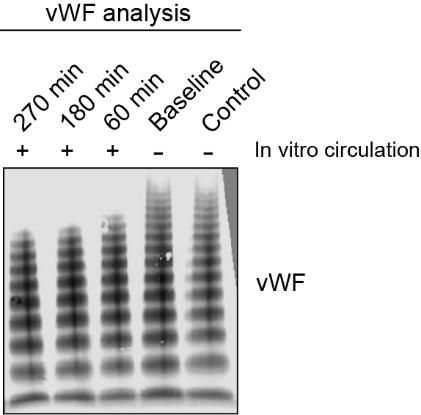


Supplemental Figure 1. Exemplary illustration of a VWF multimer analysis

with loss of the HMW multimer during circulation

Supplemental Figure 2. Comparison of the two measurement methods: impedance and fluorescence.

1: Maximum volume shift. 2: Platelet distribution width (PDW-SD). 3: Area of Fragmentocytes. 4: Area of immature platelets."*"=p<0,05. "***"=p<0,001.


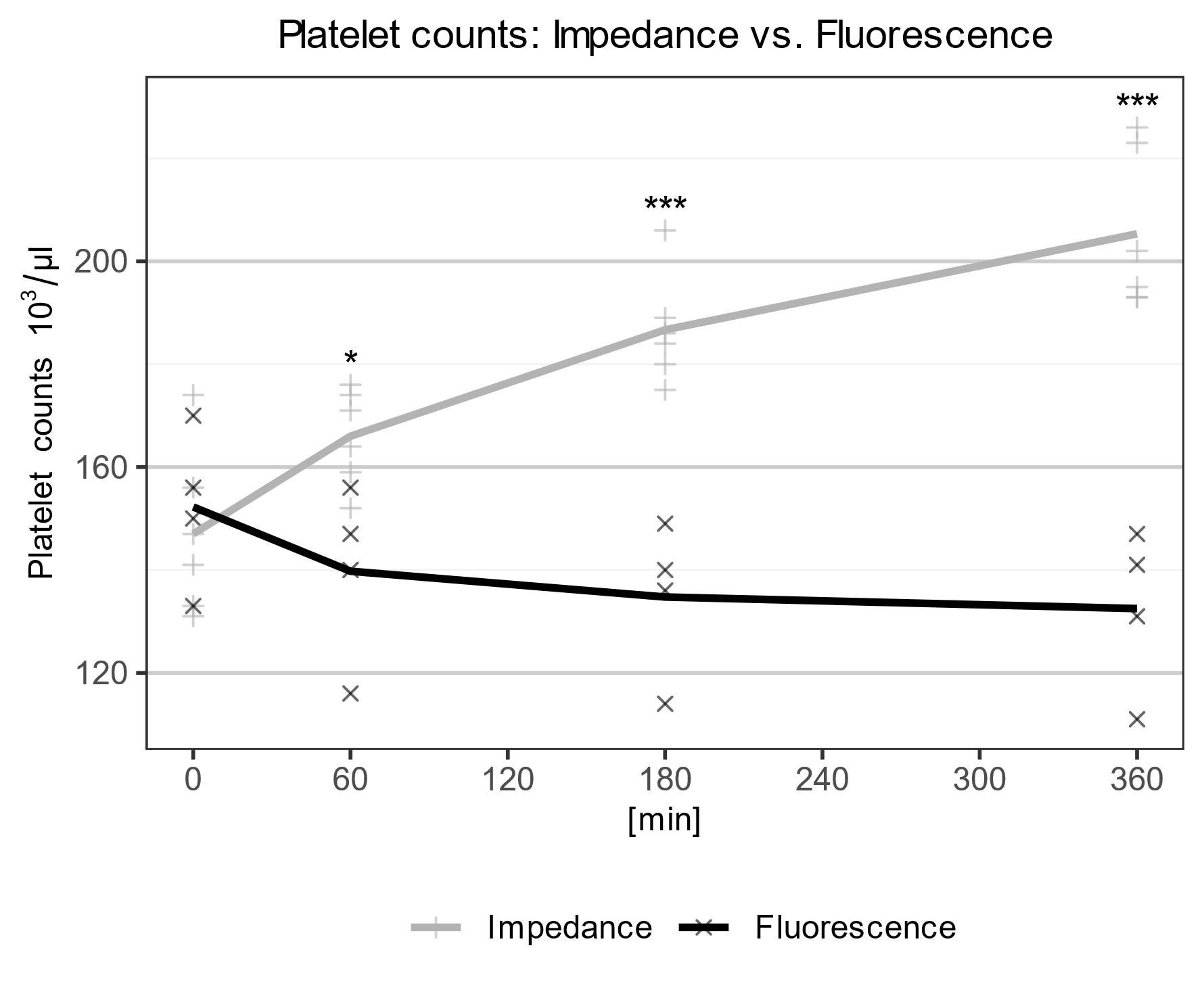


Supplemental Figure 3. Line chart with the comparison of the two measurement methods: impedance and fluorescence
